# Supplementary figures and images for: Identification of human glucocorticoid response markers using integrated multi-omic analysis from a randomized crossover trial
Source: eLife. 2021 Apr 6;10:e62236. doi: 10.7554/eLife.62236 (PMC8024021; doi:10.7554/eLife.62236)

## CONSORT 2010 Flow Diagram

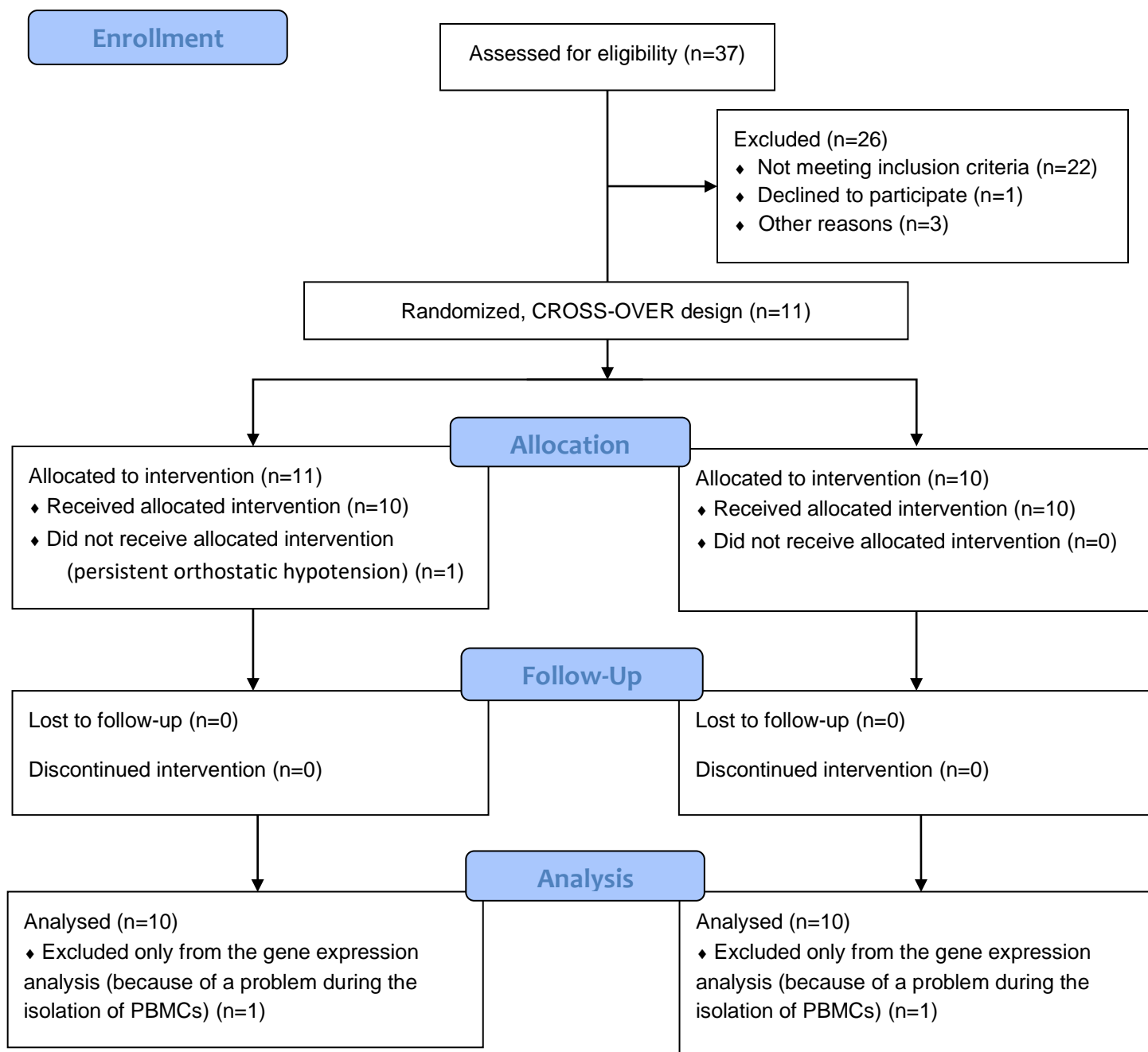

Supplement: Reporting standard 2. [file elife-62236-repstand2.pdf]
